# IslandCafe: Compositional Anomaly and Feature Enrichment Assessment for Delineation of Genomic Islands

**DOI:** 10.1534/g3.119.400562

**Published:** 2019-08-06

**Authors:** Mehul Jani, Rajeev K. Azad

**Affiliations:** * Department of Biological Sciences and BioDiscovery Institute and; † Department of Mathematics, University of North Texas, Denton, Texas 76203

**Keywords:** genomic island, horizontal gene transfer, mobile elements, segmentation, clustering

## Abstract

One of the evolutionary forces driving bacterial genome evolution is the acquisition of clusters of genes through horizontal gene transfer (HGT). These genomic islands may confer adaptive advantages to the recipient bacteria, such as, the ability to thwart antibiotics, become virulent or hypervirulent, or acquire novel metabolic traits. Methods for detecting genomic islands either search for markers or features typical of islands or examine anomaly in oligonucleotide composition against the genome background. The former tends to underestimate, missing islands that have the markers either lost or degraded, while the latter tends to overestimate, due to their inability to discriminate compositional atypicality arising because of HGT from those that are a consequence of other biological factors. We propose here a framework that exploits the strengths of both these approaches while bypassing the pitfalls of either. Genomic islands lacking markers are identified by their association with genomic islands with markers. This was made possible by performing marker enrichment and phyletic pattern analyses within an integrated framework of recursive segmentation and clustering. The proposed method, IslandCafe, compared favorably with frequently used methods for genomic island detection on synthetic test datasets and on a test-set of known islands from 15 well-characterized bacterial species. Furthermore, IslandCafe identified novel islands with imprints of likely horizontal acquisition.

Genomic islands (GIs) are clusters of functionally related genes that are mobilized across organisms through mechanisms other than vertical inheritance, namely, transformation, conjugation, transduction, cell fusion, and gene transfer agents (Juhas *et al.* 2009; Soucy, Huang, & Gogarten 2015). Horizontal gene acquisitions enable microbes to modulate their repertoire of genes. GIs are an important instrument that plays a major role in the evolution of microorganisms. GIs may be involved in adaptability, fitness and competitiveness (Dobrindt *et al.* 2003; Dobrindt *et al.* 2004), and may be important clinically as these can be enriched in genes that can lead to emergence of new pathogens or antibiotic resistant strains.

Because of the versatile functions of GIs and their impact on the evolution of microbes and on the interactions between microbes and their hosts, significant efforts have g7one into quantification of the presence of GIs and understanding of their impact on the microbial evolution (Hacker & Carniel 2001; Juhas *et al.* 2009; Langille, M. G., Hsiao, & Brinkman 2008). GIs have traditionally been deciphered using wet lab approaches that utilize subtractive hybridization or suppression subtractive hybridization, or DNA-DNA hybridization, enabling genome comparison to identify strain specific sequences (Dobrindt *et al.* 2004; Winstanley 2002). Another approach called island probing (Reyrat, Pelicic, Gicquel, & Rappuoli 1998) uses counterselectable markers (Dobrindt *et al.* 2004). However, exhaustive quantification of GIs using wet lab techniques is not feasible, in particular of the GIs that could have integrated into the genomes of close relatives. Furthermore, this could be time consuming and expensive. Whole genome sequencing and comparative genomics, spurred by the recent advances in sequencing, have enabled high-throughput genome-wide studies to localize mobile genomic elements and quantify their influence on microbial evolution. A number of computational methods that perform either standalone (single genome) or comparative (multiple genomes) analyses to identify GIs have been developed in recent years (Che, Wang, Fazekas, & Chen 2014; de Brito, Maracaja-Coutinho, de Farias, Batista, & do Rêgo 2016; Ganesan, Rakitianskaia, Davenport, Tummler, & Reva 2008; Hsiao, W., Wan, Jones, & Brinkman 2003; Langille *et al.* 2008; Langille, M. G. & Brinkman 2009; Pundhir, Vijayvargiya, & Kumar 2008; Vernikos & Parkhill 2006; Waack *et al.* 2006; Wang, Fazekas, Booth, Liu, & Che 2011; Wei *et al.* 2017). Among the most frequently used approaches are those that search for characteristic markers, such as tRNA genes in the host genome where GIs may integrate, direct repeats that flank them, integrase genes, transfer genes, phage-related sequences, conjugative elements, and other mobile genetic elements such as IS elements. Integration of GI splits the tRNA gene but is regenerated by the island sequences; GI boundaries could thus be localized by identifying the regenerated tRNA and its displaced fragment using sequence alignment (Hsiao *et al.* 2003; Reiter, Palm, & Yeats 1989). Further, GIs often possess genes involved in mobility and integration/recombination, such as those encoding integrases, transposases, recombinases and excisionases (Doublet *et al.* 2003; Langille *et al.* 2010; Osborn & Böltner 2002). Sequences flanking GIs may also bear evidence of GI integration. Phage insertion at tRNA sites often results in direct repeat sequences (Langille *et al.* 2010). GIs may also harbor genes involved in pathogenicity and drug resistance, and may be enriched in genes with yet unknown functions (“hypothetical proteins”) or of phage origin (Langille *et al.* 2010). However, this approach gives a conservative estimate of islands, missing those that integrate at sites other than tRNA or tmRNA genes, or missing islands deficient in markers that might have been eliminated by subsequent evolutionary decay of the islands.

Another class of methods exploits the atypical compositional features of GIs, such as unusual GC content or oligonucleotide composition or codon usage pattern. These atypical features reflect the compositional biases of the donors and can thus be identified as distinct signals against the recipient genome background. These methods often invoke either bottom-up gene by gene approach, *e.g.*, SIGI-HMM that examines codon usage bias, (Waack *et al.* 2006; Wei *et al.* 2017) or moving window approach, *e.g.*, AlienHunter and SeqWord that use oligomer frequencies within a sliding window (Vernikos & Parkhill 2006) and Mean Shift Genomic Island Predictor (MSGIP) that detects windows of genes with atypical composition (de Brito *et al.* 2016). Because of the variable compositional character of GIs, weakly atypical genes or windows are often misclassified, which complicates the detection of island boundaries. Furthermore, the moving window methods are sensitive to window size– smaller size increases stochastic fluctuations while larger size diminishes resolution. These methods are also prone to misclassifying genes or regions that display anomalous composition for reasons other than horizontal acquisition.

To minimize false positives, combinations of these approaches have also been proposed. IslandPath-Dimob uses presence of mobility genes, t-RNA insertion sites, and dinucleotide compositional bias to predict GIs (Hsiao *et al.* 2003). IslandViewer (Bertelli *et al.* 2017; Langille & Brinkman 2009) is an integrated interface that combines the predictions of different methods including SIGI-HMM and IslandPath-Dimob to localize GIs with high confidence. GIHunter (Wang *et al.* 2011) uses eight GI-associated features including gene density, intergenic distance, phage genes, tRNA genes, genes encoding integrase or transposase, highly expressed genes, and the AlienHunter score. These methods could be highly specific, though at the cost of numerous false negatives. In contrast, PredictBias (Pundhir *et al.* 2008), a moving window approach, is designed to be more sensitive- genomic regions with unusual codon usage and either atypical GC composition or atypical dinucleotide composition, or regions with an abundance of virulence genes, are deemed GIs.

Among the comparative genomics or phylogenetic methods, one approach is to search for genomic regions with limited phylogenetic distribution, that is, those regions that are absent from the genomes of close relatives are inferred as GIs. IslandPick (Langille *et al.* 2008) is an example of this class of methods. This approach, however, requires multiple strains of closely related species. Lineage-specific gene loss may further confound the inference.

It was recently suggested that GIs could be more robustly identified using a top-down approach that allows examination of genes *en mass*e (Arvey, Azad, Raval, & Lawrence 2009; Azad, R. K. & Li 2013). The premise of this approach is simultaneous analysis of GI harbored genes, both weakly atypical and strongly atypical, in order to decipher the GI structure more robustly. One such method, MJSD, which is based on a recursive segmentation procedure, indeed delineated GIs significantly better than other methods (Arvey *et al.* 2009). This has spurred development of more segmentation based methods, including Zisland Explorer that uses cumulative GC profile for GI identification (Wei *et al.* 2017) and GEMINI that utilizes segment context information within an integrated segmentation and clustering framework to delineate GIs.

Although further development of more sensitive, top-down methods for GI detection is sorely needed, there are significant challenges that must be overcome. The segmentation procedure is often followed by an agglomerative clustering procedure for grouping of compositionally similar segments (Azad & Li 2013). This makes possible identification of “typical” (the most abundant, potentially vertically inherited or native) and “atypical” (potentially horizontally acquired or alien) genomes by virtue of the cluster size, with the largest cluster representing the genome backbone (*i.e.*, the native genome) while the others representing the accessory or alien genome. Although this makes possible an unbiased analysis of a genome, agglomerative clustering of segments is fraught with risks. Grouping of segments at stricter stringencies may render clusters that are “pure”, *i.e.*, harboring either native or alien DNA segments but not both, however, numerous native clusters as well as alien clusters thus generated could complicate the reconstruction of the genome structure. Relaxing the stringency may result in undesirable mergers of clusters. This is particularly true in this case where weakly typical, native segments may coalesce into one or more clusters that are deemed distinct from the cluster harboring strongly typical, native segments; allowing these native clusters to merge by relaxing the stringency may cause clusters harboring weakly atypical, alien segments to coalesce with the native clusters. This may also result in undesirable mergers of clusters representing closely related but distinct donors. On the other hand, certain class of native segments, *e.g.*, those harboring highly expressed genes of apparently unusual codon usage, may coalesce into clusters of their own and may not merge with the backbone cluster even at relaxed stringencies. One way to circumvent this is to utilize biological information, such as segment context information, for cluster merger after generating apparently pure clusters at a stringent clustering threshold (Jani, Mathee, & Azad 2016). Further experiments show that whereas this may work for some strains, this is not universally applicable for any choice of threshold (further discussed below).

Deconstruction of segmental structure underlying a genome is critical to deciphering GIs, as the data from recent studies suggest (Azad & Li 2013; Wei *et al.* 2017). Furthermore, for a sensitive and robust GI detection, GIs that have lost the identifying features or markers must also be identified and atypical segments that are compositionally deviant for reasons other than HGT must be eliminated from predictions. The success of such procedures depends on robust grouping of genes or segments of similar ancestry or origin. In our survey of the methods for GI detection, we observed that none of the current methods has the ability to accomplish this. We therefore embarked on developing an approach that could utilize biological information specific to GIs within an integrated framework of segmentation and clustering. We hypothesized that GIs lacking the identifying features or markers could be identified if analyzed together with GIs enriched in these markers. Our hypothesis is based on the assumption that not all GIs originating from a donor taxon would have lost many or most identifying markers contemporaneously, and if this is true, GIs deficient in markers could be identified by virtue of their association (*i.e.*, origin) with GIs enriched in markers. This association could be explored in a number of ways; here, we infer the association based on similar compositional bias shared by GIs originating from a specific donor taxon. Based on this premise, after obtaining clusters at a stringent threshold, if a cluster is found enriched in GI specific markers (assessed against whole genome background), all segments within the cluster, whether enriched or not in markers, are deemed GIs (or, parts of GIs or the alien genome). On the other hand, if a cluster is not enriched in GI specific markers, all segments within the cluster are deemed “non-GIs”, provided genes harbored by these segments do not display aberrant phyletic pattern. We tested our hypothesis on synthetic test datasets and on a comprehensive dataset of known genomic islands from 15 well-characterized bacterial species. Our method, IslandCafe, named after detection of Island by Compositional anomaly and feature enrichment, compared favorably with frequently used methods for genomic island detection. In what follows, we further elaborate on our method and the genomes that were used for assessment, present our results, and conclude with a discussion.

## Materials and Methods

### Genome sequences and annotations

The complete genome sequences and annotations of 15 bacteria representing a host of taxa, namely, *Acinetobacter baumannii* AYE (NC_010410.1), *Bartonella tribocorum* CIP 105476 (NC_ 010161.1), *Bordetella petrii* DSM 12804 (NC_010170.1), *Burkholderia cenocepia* J2315 (NC_011000.1, NC_011001.1 and NC_011002.1), *Burkholderia pseudomallei* K96243 (NC_006350.1 and NC_006351.1), *Clavibacter michiganensis* NCPPB 382 (NC_009480.1), *Corynebacterium diptheriae* NCTC 13129 (NC_002935.2), *Escherichia coli* CFT073 (NC_004431.1), *Mesorhizobium loti* MAFF 303099 (NC_002678.2), *Proteus mirabilis* HI4320 (NC_010554.1), *Pseudomonas aeruginosa* LESB58 (NC_011770.1), *Salmonella enterica serovar Typhi* CT18 (NC_003198.1), *Staphylococcus aureus* USA300 FPR3757 (NC_007793.1), *Streptococcus equi* 4047 (NC_012471.1), and *Vibrio cholerae* O1 biovar eltor str. N16961 (NC_002505.1 and NC_002506.1) were obtained from NCBI FTP site (ftp://ftp.ncbi.nlm.nih.gov/genomes/archive/old_genbank/Bacteria/). These bacteria were previously well-studied for the presence of genomic islands; genomic island coordinates and the supporting evidence from these studies are provided in Table S1.

### Synthetic genomes

In addition to the aforementioned genomes, synthetic genomes were constructed for assessment for IslandCafe and other methods. *Burkholderia cenocepacia* J2315 was selected as the recipient organism. All GI prediction methods considered in this study were then applied to catalog putative GIs in *B. cenocepacia* J2315s primary chromosome (chromosome 1). *B. cenocepacia* J2315 genomic sequences that were not predicted as GIs by any method were extracted and concatenated. This provided a conservative backbone (core) genome of *B. cenocepacia* J2315. Backbone genomes were obtained similarly for selected donors, namely *Pseudomonas aeruginosa* LESB58 and *Stenotrophomonas maltophilia* D457 (secondary chromosomes, where present, were not considered for extraction of the backbone genomes). A synthetic genome was constructed by simulating transfer of segments from the backbone genomes of donors into the *B. cenocepacia* J2315 backbone genome. 12 Segments of size 30 Kbp, 50 Kbp, and 80 Kbp were randomly extracted from the backbone genome of each donor and inserted into the *B. cenocepacia* backbone genome; these GIs in a synthetic genome represented ∼16% of the original *B. cenocepacia* J2315 chromosome 1. Multiple synthetic genome replicates were thus obtained and three test datasets were then generated by randomly introducing marker genes from donor genomes in 25%, 50%, and 75% of the GIs in synthetic genomes, respectively. It is possible that some of the sequences sampled from donor backbone genomes for insertion might already have one or more marker genes, so the GIs in the synthetic genome selected for marker introduction contain at least one marker gene, following insertion of a marker gene (transposase) into each of these GIs.

### Current genomic island prediction tools

We assessed IslandCafe against ten frequently used GI prediction methods, namely, IslandPick (Langille *et al.* 2008), GIHunter (Che *et al.* 2014), IslandPath-Dimob (Hsiao *et al.* 2003), MSGIP (de Brito *et al.* 2016), SIGI-HMM (Waack *et al.* 2006), Zisland Explorer (Wei *et al.* 2017), AlienHunter (Vernikos & Parkhill 2006), PredictBias (Pundhir *et al.* 2008), SeqWord (Ganesan *et al.* 2008), and IslandViewer (Langille & Brinkman 2009). These include both well-established and recently developed methods. MSGIP, SIGI-HMM, Zisland Explorer, AlienHunter, and SeqWord search for regions with anomalous codon usage and/or oligonucleotide composition. IslandPath-Dimob and GIHunter search for regions harboring GI specific markers and combine this with compositional signatures to predict GIs. PredictBias bases its prediction on compositional bias; in addition, it outputs segments that are enriched in virulence genes. IslandPick identifies putative GIs via sequence comparison, and IslandViewer combines predictions by three different methods, namely, SIGI-HMM, IslandPath-DIMOB, and IslandPick.

### Compositional anomaly assessment

Our proposed method, IslandCafe, combines compositional anomaly, a hallmark of GIs, with the functional or structural features that characterize GIs within the framework of a recursive segmentation and agglomerative clustering procedure. This integrative framework allows fragmentation of a genome into compositionally homogeneous segments that are then segregated into clusters reflecting their potentially shared ancestries (Azad & Li 2013; Jani & Azad 2013). The binary segmentation process entails division of a genome into two segments at a position where the compositional difference between the resulting segments is maximum, provided this difference is deemed statistically significant. The compositional difference is quantified using an information-entropy based divergence measure, namely, Jensen-Shannon divergence generalized within the framework of Markov chain model of order m, defined as (Arvey *et al.* 2009; Thakur, Azad, & Ramaswamy 2007),

$$D^{m}\left(p_{1},p_{2}\right)=H^{m}\left(\pi_{1}p_{1}+\pi_{2}p_{2}\right)-\pi_{1}H^{m}\left(p_{1}\right)-\pi_{2}H^{m}(p_{2}).$$

$D^{m}(p_{1},\;p_{2}$) denotes the divergence between two sequence segments represented by the respective probability distributions *p*_1_ and *p*_2_, each comprising the marginal and conditional (transitional) probability distributions. $H^{m}\left(.\right)$ is the entropy function for Markov source of order m, defined as, $H^{m}\left(p_{i}\right)=-\underset{w}{\sum }P\left(w\right)\underset{x\in A}{\sum }P\left(x\text{|}w\right)\log_{2}P\left(x\text{|}w\right)$. P(x|w) is the probability for transitioning from oligonucleotide w to the succeeding nucleotide x,and P(w) is the probability of oligonucleotide w of length *m* that defines that order of the Markov model. $\pi_{i}$ is the weight factor assigned to *p_i_*.

The statistical significance of a value of the divergence measure is assessed using the probability distribution of this measure in random sequences, which has been shown to approximate the chi-square distribution function (Arvey *et al.* 2009; Grosse *et al.* 2002),

$P\left(D^{m}\leq X\right)\approx \chi_{v}^{2}\left(2L\left(ln2\right)X\right),$ where, $v=k^{m}\left(k-1\right)$ is the degrees of freedom, with alphabet size *k* = 4 for DNA sequences, and *L* is the length of the sequence to be segmented. Probability distribution of the maximum value of this measure has been shown to approximate chi-square distribution function with fitting parameters (Arvey *et al.* 2009; Grosse *et al.* 2002; Thakur *et al.* 2007):

$$P(D_{max}^{m}\leq X)\approx {\left\{\chi_{v}^{2}\left[2L\left(ln2\right)X\beta \right]\right\}}^{N_{\text{eff}}},$$

*β* and $N_{\text{eff}}$ are the fitting parameters whose values are estimated by fitting the above equation to the empirical distributions obtained via Monte Carlo simulations. The recursion of segmentation is halted when none of the segments can be divided further. Since hyper-segmentation is allowed for precise delineation of GI boundaries, an agglomerative clustering of similar segments is performed at a relatively relaxed clustering stringency in two steps (Azad & Li 2013). In the first step, compositionally similar, contiguous segments are identified and merged, thus restoring the underlying segmental structure. The second clustering step allows grouping of compositionally similar clusters recursively, performed within the same framework of statistical hypothesis testing (Azad & Li 2013). This procedure, by virtue of its ability to segregate segments of different lineages, recovers the backbone (vertically inherited) genome as the largest cluster (typically with more than half of the genome), whereas smaller clusters represent the potential donor taxa (Garcia-Vallve, Guzman, Montero, & Romeu 2003; Lawrence & Ochman 1998). A significant challenge, however, lies here in classifying the segments by their origins after the segmental structure of a genome is uncovered (*i.e.*, after the first clustering step). Often, native segments are grouped into two or more clusters, with the largest harboring the strongly typical native segments. The weakly typical native segments tend to form cluster(s) of their own. Relaxing the statistical threshold to allow merger of these clusters could also result in merger of one or more alien clusters with the native clusters. Robust merger of clusters is critical to the success of this class of methods as any cluster-level misclassification renders all segments within the cluster misclassified. By combining this statistical framework with GI specific marker enrichment and phyletic pattern assessment, we show here that this problem can be satisfactorily addressed.

### Feature enrichment and phyletic pattern analysis

After generating clusters at a stringent clustering threshold within the statistical hypothesis testing framework, we first perform an enrichment analysis to identify clusters enriched in features or markers that are typically associated with GIs. We posit that weakly typical, native clusters are not enriched in these features, whereas alien clusters, both strongly atypical and weakly atypical, are enriched. IslandCafe identifies markers that typically characterize GI (Table S2) by parsing the input genome annotation file. Given a genome sequence, IslandCafe compiles markers by first employing Prodigal (Hyatt *et al.* 2010) to predict genes and then HMMER (Eddy 1998; Finn, Clements, & Eddy 2011) to annotate markers. A custom Pfam HMM database was created by performing search for GI markers using keywords such as transposase in the Pfam database (Finn *et al.* 2015). The custom database was manually curated to eliminate any profile HMMs not representing GI markers. This resulted in a local database of 449 profile HMMs representing marker gene families (Table S3). Genes from a genome of interest are probed against this database and those with “hits” in the database with expect values 0.01 or less (Hsiao *et al.* 2005) are annotated as marker genes.

**Figure 1:**
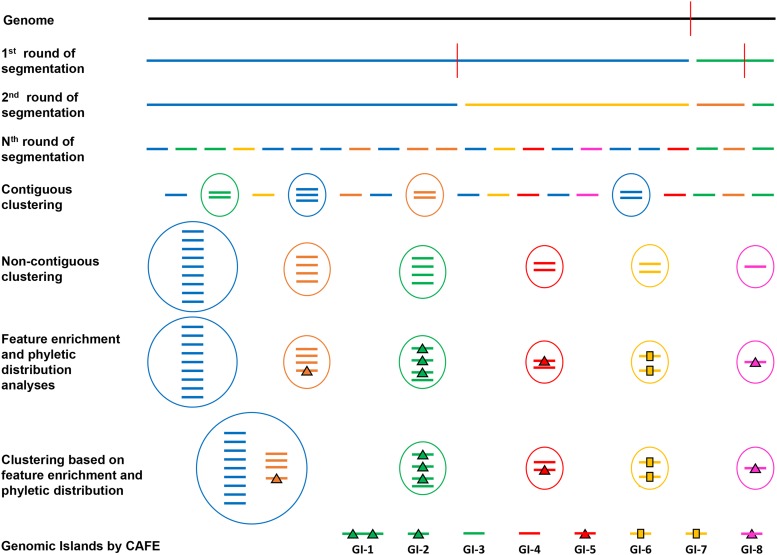
Schematic representation of IslandCafe’s protocol for identifying genomic islands. A genome is divided recursively into compositionally homogeneous segments (“Segmentation”, here each segment is shown by a colored horizontal thick line; the red vertical line indicates position where the divergence is maximum). “Contiguous clustering” is the first round of agglomerative clustering procedure, which entails identification and merger of compositionally similar contiguous segments (contiguous similar segments are shown in a circle). “Non-contiguous clustering” is the second round of agglomerative clustering procedure, which entails recursive grouping of compositionally similar segments, including non-contiguous similar segments (single lines and groups of lines (circles) of same color are now merged into a single cluster). “Feature enrichment and phyletic distribution analyses” entails identification of GI specific features in each segment (shown as Δ on a segment) and identification of genes with aberrant phyletic distribution (shown as on a segment). “Clustering based on feature enrichment and phyletic distribution” involves identification and merger of native clusters (blue and orange clusters that are not enriched in GI specific markers or lack genes with aberrant phyletic distribution) while precluding undesirable mergers of alien clusters. IslandCafe calls any segments or chains of contiguous segments, 8 Kbp or greater, belonging to alien clusters as GIs.

Following segmentation and clustering performed for each genome, parts of the native or backbone genome are first identified by the largest cluster; the smaller clusters are then examined and processed as follows. If a cluster is found enriched in marker genes (one and half fold relative to the genome), it is deemed an alien cluster. Non-enriched clusters are deemed alien only if their genes display aberrant phyletic pattern, *i.e.*, absence from majority of the close relatives of the genome harboring the gene of interest. Aberrant phyletic distribution is assessed by aligning the sequence of the protein encoded by the gene of interest against all protein sequences originating from the same taxon as the gene of interest using BLASTP (Altschul, Gish, Miller, Myers, & Lipman 1990). A custom database of protein sequences for all bacteria at the NCBI ftp site (ftp://ftp.ncbi.nlm.nih.gov/genomes/archive/old_genbank/Bacteria/) was constructed to accomplish this. If a query protein returns BLAST hits with >70% identity and query coverage in less than half of the organisms from the same genus as the organism with the query sequence, the gene encoding the query protein is deemed phyletically atypical or aberrant. We thus identify weakly typical, smaller native clusters based on enrichment and phyletic pattern, that is, those lacking marker enrichment and aberrant phyletic distribution, and merge them to the largest cluster harboring the strongly typical native segments. The remaining clusters, enriched in markers or displaying atypical gene distribution, are deemed alien. GIs are identified as contiguous alien segments, 8 Kbp or more in size (Schmidt & Hensel 2004). An illustrative diagram of the pipeline implemented in IslandCafe is shown in Figure 1.

### Performance assessment

Comparative assessment of IslandCafe was performed against ten frequently used GI prediction methods at both nucleotide-level (*i.e.*, ability to identify GI nucleotides) and island-level (*i.e.*, ability to identify a GI as a single segment or a chain of contiguous segments). In assessing the island-level accuracy, if a method predicts several segments spanning a known GI, only the largest overlapping segment is considered. If the largest overlapping segment is more than twice the size of the corresponding GI, it is not considered a true prediction. The standard performance metrics, namely, recall (or sensitivity), precision, average performance, F-measure, performance coefficient, and Matthews correlation coefficient (MCC), as defined below, were used for performance assessment.

$$Recall=\frac{TP}{TP+FN}$$

$$Precision=\frac{TP}{TP+FP}$$

$$Average\;Performance=\frac{Recall+Precison}{2}$$

$$F-measure=\frac{2\cdot Precision\cdot Recall}{Precision+Recall}$$

$$Performance\;Coefficient=\frac{TP}{TP+FP+FN}$$

$$MCC=\frac{TP\cdot TN-FP\cdot FN}{\sqrt{\left(TP+FP)(TP+FN)(TN+FP)(TN+FN\right)}}$$

Here TP, TN, FP, FN are the numbers of true positives, true negatives, false positives, and false negatives respectively. All these performance metrics have values lying between 0 and 1, which are shown either as fraction or percentage in the following sections. Together these metrics quantify the accuracy of a method in identifying GIs or discriminating GIs from non-GIs. Note that we didn’t consider Classification Accuracy, defined as (TP+TN)/(TP+TN+FP+FN), for assessment here, as this is not an appropriate metric for an unbalanced dataset, which is the case here for both synthetic and datasets; *e.g.*, real dataset has ∼91% non-GI nucleotides and only ∼9% GI nucleotides. As the number of GI nucleotides is much smaller (∼6.6 Mb across all 15 species) than the nucleotides in the rest of the genomes (∼69.7 Mb), a method that identifies only few or even no GI nucleotides will have a Classification Accuracy of ∼90%. In contrast, the accuracy measures such as Recall and Precision and their combination, the F-measure, are considered appropriate for unbalanced datasets.

### Data Availability

IslandCafe is an open source collaborative initiative available in the GitHub repository (https://github.com/mehuljani/IslandCafe). The following data are listed in the supplementary table: Evidence from the literature supporting presence of GIs in the genomes used for this study (Table S1), list of genes often associated with GIs (Table S2), list of PFAM ids used to make database for identifying GI specific features or marker genes (Table S3), performance of segmentation and clustering algorithm in identifying GIs in 15 bacterial species at various segmentation and clustering thresholds (Table S4), performance of segmentation and clustering algorithm after including marker gene enrichment and phylogenetic modules (Table S5), performance of GI prediction tools in identifying nucleotides belonging to GIs and identifying GIs in synthetic *B.cenocepacia* genome dataset with 25% of all GIs harboring marker genes (Table S6), performance of genomic island prediction tools in identifying nucleotides belonging to GIs and identifying GIs in synthetic *B. cenocepacia* genome dataset with 50% of all GIs harboring marker genes (Table S7), performance of genomic island prediction tools in identifying nucleotides belonging to GIs and identifying GIs in synthetic *B. cenocepacia* genome dataset with 75% of all GIs harboring marker genes (Table S8), performance of genomic island prediction tools in classifying nucleotides as belonging to GIs and non-GIs for 15 bacterial species (Table S9), performance evaluation of genomic island prediction tools for identifying islands with at least 75% overlap between the predicted segment and the corresponding known island in 15 bacterial species (Table S10), performance evaluation of genomic island prediction tools for identifying islands with at least 95% overlap between the predicted segment and the corresponding known island in 15 bacterial species (Table S11), performance of GI prediction methods in identifying GIs in 15 bacterial species at 50% cutoff of overlap between the predicted segment and the corresponding known GI (Table S12), performance of GI prediction methods (assessed by F-measure) in identifying GIs in 15 bacterial species at 75% cutoff of overlap between the predicted segment and the corresponding known GI (Table S13), performance of GI prediction methods (assessed by F-measure) in identifying GIs in 15 bacterial species at 95% cutoff of overlap between the predicted segment and the corresponding known GI (Table S14), coordinates of GIs predicted by CAFE in the 15 representative genomes (Table S15), and grouping of GIs lacking marker genes with GIs harboring marker genes arising from a donor within a distinct cluster (Table S16). Supplemental material available at FigShare: https://doi.org/10.25387/g3.9255608.

## Results

### Segmentation and clustering in GI detection

An integrated segmentation and clustering approach was shown to work well in deciphering GIs in bacterial genomes (Azad & Li 2013). Comparative assessment with other methods on an artificial chimeric *E. coli* genome and a well-understood *Salmonella enterica typhi* CT18 genome demonstrated the power of this method in delineating large structures such as GIs in bacterial genomes (Azad & Li 2013). Here, we assessed the same approach on a broader dataset, including 15 different species as listed in Materials section above. Both segmentation and clustering thresholds were varied. Segmentation threshold (significance level) was varied from 1E-2 to 1E-20, and similarly, the clustering thresholds were varied in this range for each segmentation threshold to identify the optimal setting of the program (Table S4). The highest accuracies on the combined test set of GIs and non-GIs from all 15 species were 0.55, 0.34, and 0.50 when considering average performance, performance coefficient, and F-measure respectively as the overall accuracy parameter (Table 1). Results for individual genomes show that the optimal performance was obtained at different threshold settings for these genomes, which may differ with the optimal threshold setting for the entire dataset (Table 1). This suggests that a “universal” optimal parameter setting is difficult to realize within this framework.

**Table 1:** Performance of segmentation and clustering algorithm in identifying GI nucleotides in 15 representative genomes. The values of performance metrics are shown for the optimal threshold setting for each genome and also for the optimal setting for the entire test dataset (all 15 genomes individually run at same thresholds). Contiguous clustering threshold refers to the significance threshold used in the first round of agglomerative clustering procedure to identify and merge compositionally similar contiguous segments. Non-contiguous clustering threshold refers to the significance threshold used in the second round of agglomerative clustering procedure to recursively group compositionally similar segments, including non-contiguous similar segments

Genome	Segmentation threshold	Contiguous clustering threshold	Non-contiguous clustering threshold	Recall	Precision	Average Performance	Performance Coefficient	F-measure	MCC
*Acinetobacter baumannii* AYE	10^−1^	10^−10^	10^−10^	0.60	0.64	0.62	0.45	0.62	0.61
*Bartonella tribocorum* CIP 105476	10^−3^	10^−3^	10^−4^	0.75	0.40	0.58	0.35	0.52	0.28
*Bordetella petrii* DSM 12804	2·10^−1^	10^−2^	10^−4^	0.99	0.69	0.84	0.69	0.82	0.78
*Burkholderia cenocepia* J2315	10^−1^	10^−7^	10^−10^	0.92	0.45	0.68	0.41	0.58	0.60
*Burkholderia pseudomallei* K96243	10^−2^	10^−10^	10^−6^	0.73	0.47	0.60	0.39	0.56	0.55
*Clavibacter michiganensis* NCPPB 382	10^−3^	10^−10^	10^−10^	0.55	0.71	0.63	0.45	0.62	0.61
*Corynebacterium diptheriae* NCTC 13129	10^−3^	10^−4^	10^−5^	0.26	0.87	0.57	0.25	0.40	0.45
*Escherichia coli* CFT073	10^−3^	10^−10^	10^−5^	0.86	0.71	0.78	0.63	0.77	0.73
*Pseudomonas aeruginosa* LESB58	2·10^−1^	10^−7^	10^−4^	0.91	0.38	0.65	0.37	0.53	0.55
*Mesorhizobium loti* MAFF 303099	10^−1^	10^−10^	10^−10^	0.94	0.53	0.73	0.51	0.68	0.67
*Staphylococcus aureus* USA 300	10^−2^	10^−2^	10^−2^	0.89	0.16	0.53	0.16	0.28	0.26
*Proteus mirabilis* HI4320	10^−3^	10^−10^	10^−3^	0.55	0.60	0.57	0.40	0.57	0.53
*Salmonella enterica* Typhi CT18	10^−1^	10^−10^	10^−10^	0.74	0.57	0.65	0.47	0.64	0.61
*Streptococcus equi* 4047	10^−2^	10^−10^	10^−6^	0.99	0.22	0.61	0.22	0.36	0.35
*Vibrio cholerae* O1 biovar eltor str. N16961	10^−4^	10^−2^	10^−3^	0.97	0.85	0.91	0.83	0.91	0.90
All genomes^†^	10^−1^	10^−10^	10^−7^	0.72	0.39	0.55	0.34	0.50	0.47

† All genomes means the combined test set of GIs and non-GIs from all 15 species. TP, TN, FP, and FN for the combined set denote the respective aggregate values from all 15 species. That is, each of these was first obtained for each species and then combined for all 15 species before computing the values of performance metrics for the combined test set.

We further analyzed these genomes using the current version of this program that additionally used the segment context information (Jani *et al.* 2016) to improve GI detection in *Pseudomonas aeruginosa* LESB58. This strain was previously reported to harbor a number of pathogenicity and resistance islands (Winstanley, C. *et al.* 2009). As the genome of *Pseudomonas aeruginosa* LESB58 is well-characterized, with at least four GIs experimentally validated, the cluster configurations for this genome were earlier examined at different threshold settings, from a stringent setting generating numerous pure clusters to a relaxed setting generating hybrid clusters (harboring both native and alien segments) in addition to the pure clusters. Simultaneous formation of two large native clusters was observed, the largest with ∼62.5% of the genome harbored strongly typical, native segments and the other with ∼24.7% of the genome harbored compositionally ambiguous (weakly typical) native segments, whereas the third largest cluster with ∼7.1% of the genome harbored alien segments. Numerous smaller clusters were also formed in the process. Attempt to merge the large native clusters by successively relaxing the clustering stringency results in the merger of the largest alien cluster with the largest (native) cluster. This highlights the difficulty in segregating native and alien segments based on statistical approach alone. Earlier we had proposed utilizing segment context information to circumvent this limitation (Jani *et al.* 2016). This was based on the observation that compositionally ambiguous native segments are sparsely distributed within the genome. While this provided a cue to merge native clusters in the LESB58 genome, our analysis on the broader genome set showed that this approach may not work for some genomes, specifically when the clusters have too few segments to allow this statistical analysis. For example, the application of the recursive segmentation and clustering procedure to the *Vibrio cholerae O1 biovar eltor* str. N16961 Chr. 2 (1.07 Mb) genome resulted in seven clusters, however, the three largest clusters have only 2-3 segments each, precluding a reliable inference based on segment context information.

### Utilizing feature enrichment and phyletic distribution in GI detection

Although segmentation and clustering provide a powerful tool for segregating compositionally similar segments, and in the process identifying the potential GIs, grouping of compositionally distinct native clusters while precluding undesirable mergers of alien and native clusters is a major bottleneck that must be addressed in order to take the state-of-the-art in GI prediction to new heights. We posit that since it is almost impossible to accomplish this task by tinkering with the algorithmic parameters within the hypothesis testing framework, a significant advance in the field could be possible by utilizing any biological information that could complement the compositional information encoded in GIs. We therefore attempted to complement the composition-based segmentation and clustering with functional or structural information embodied in GIs and the phyletic distribution of GI harbored genes. Functional features or markers that are frequently observed in GIs include genes that are associated with integration/recombination and transposition, phage metabolism, plasmid, and insertion (Table S2). While these features have previously been used to localize GIs, here we use this information for cluster merger, particularly to merge weakly typical, native cluster(s) with the strongly typical, native cluster. We postulate that alien clusters, particularly the large ones, are replete with GIs and therefore, are enriched in the aforementioned features that characterize GIs. On the other hand, the weakly typical native clusters are expected to be depleted of GI specific features. We use this difference in the enrichment of functional/structural features in the clusters to identify weakly typical, native clusters and merge them with the strongly typical, native cluster. This also allows us to eliminate spurious predictions arising from clusters with genes that are compositionally atypical for reasons other than HGT. For example, highly expressed native genes often have an unusual codon usage or compositional bias and are therefore prone to be misclassified as alien. However, as these clusters are not enriched in GI specific features, the proposed approach classifies them as native, thus minimizing false positives.

The presence of GI specific features was quantified for each atypical cluster outputted by the segmentation and clustering algorithm, and if an atypical cluster was enriched in these features, it was deemed alien. Substantial performance improvement was observed following this procedure (Table S5). Further improvement was achieved when non-enriched atypical clusters were examined for the distribution of their genes in close relatives and deemed alien if their genes displayed aberrant phyletic pattern (Table S5).

### Comparative assessment of IslandCafe with other GI prediction methods

#### Rationale for comparative assessment at both nucleotide and island level:

Most previous studies evaluated GI prediction tools’ performance based on their ability to classify genes as GI- or non-GI-borne genes (Che *et al.* 2014; Wei *et al.* 2017). However, GIs are clusters of functionally related genes that are acquired *in toto* rather than in multiple horizontal transfers of single genes at a genomic locus. Following acquisition, a GI may again be mobilized within or across genomes as an intact unit. It is therefore important that the GI prediction methods identify islands as single structures. Often, the GI prediction methods are unable to identify GIs as single, continuous entities; the rather fragmented predictions could be missing not just the large chunks of GIs but could also be overestimating the number of GIs in a genome. On the contrary, some methods predict large structures that may overlap with only small portion of known GIs. In order to assess the ability of method to recover a known GI as a single segment or a chain of contiguous segments, we considered here, for the GI level assessment, only the predicted segment (or chain of contiguous segments) with the largest overlap with the known GI as the true prediction by a method, provided this prediction satisfies a preset criterion (see below). This allows a fair assessment of the ability of a method to reconstruct GI structure within a genome; a reasonably robust method is expected to identify a GI as a single segment (or a chain of contiguous segments) with substantial overlap with the GI and also comparable in size to the GI it overlaps with.

We evaluated IslandCafe against other GI prediction tools for localizing GIs at 50%, 75%, and 95% cutoffs, where an X% cutoff means that X% of a GI should be identified with the predicted segment (or chain of contiguous segments) in order for the prediction to be deemed a success. Thus, the GI level performance assessment was accomplished by obtaining the recall of a method as the fraction of known GIs that were correctly identified as single segments (or chains of contiguous segments) at X% cutoff, and the precision of a method as the fraction of all predicted GIs that were true predictions (known GIs) at X% cutoff. The harmonic mean of these two metrics defined the F-measure that was used as a single accuracy measure for GI-level performance assessment. Note that this evaluation procedure was followed only for GI level assessment; the nucleotide level performance evaluation was still done by assessing whether known GI and non-GI nucleotides were correctly classified using the assessment measures described in the Materials and Methods section. While the latter does not attest to a method’s ability to decipher the GI structure, it does provide information on the fraction of a genome that is deemed GIs or parts of GIs by a method. Together, these two evaluation procedures provide insights into the ability of the methods to predict GI based accessory genome and the GIs themselves. As GIs are often acquired in single evolutionary events, GI prediction also provides an estimate of the horizontal transfer events involving GIs, an important information that sheds light on evolutionary processes shaping bacterial genomes.

#### Using synthetic genomes for assessment:

We first evaluated the GI prediction methods for their ability to detect GIs in synthetic genomes. As the evolutionary history of the segments (*i.e.*, island or non-island) is known in synthetic genomes, these provide a valid dataset for evaluating GI prediction methods. The donors, *P. aeruginosa* LESB58 and *S. maltophilia* D457, represent the class Gammaproteobacteria, distinct from the class Betaproteobacteria that the recipient *B. cenocepacia* J2315 belongs to. Both the donors and the recipient *B. cenocepacia* J2315 used in constructing synthetic genomes were previously observed cohabiting the lungs of the cystic fibrosis patients (Denton, Todd, Kerr, Hawkey, & Littlewood 1998; Holden *et al.* 2009; Winstanley *et al.* 2009), and could potentially be exchanging DNAs; indeed horizontal gene transfers among bacteria cohabiting the cystic fibrosis lungs have previously been reported (Jani *et al.* 2016). The performance of the GI prediction methods was assessed on these test data, by averaging over 5 synthetic genome replicates.

#### Classification of GI and non-GI nucleotides in synthetic genomes:

**Figure 2:**
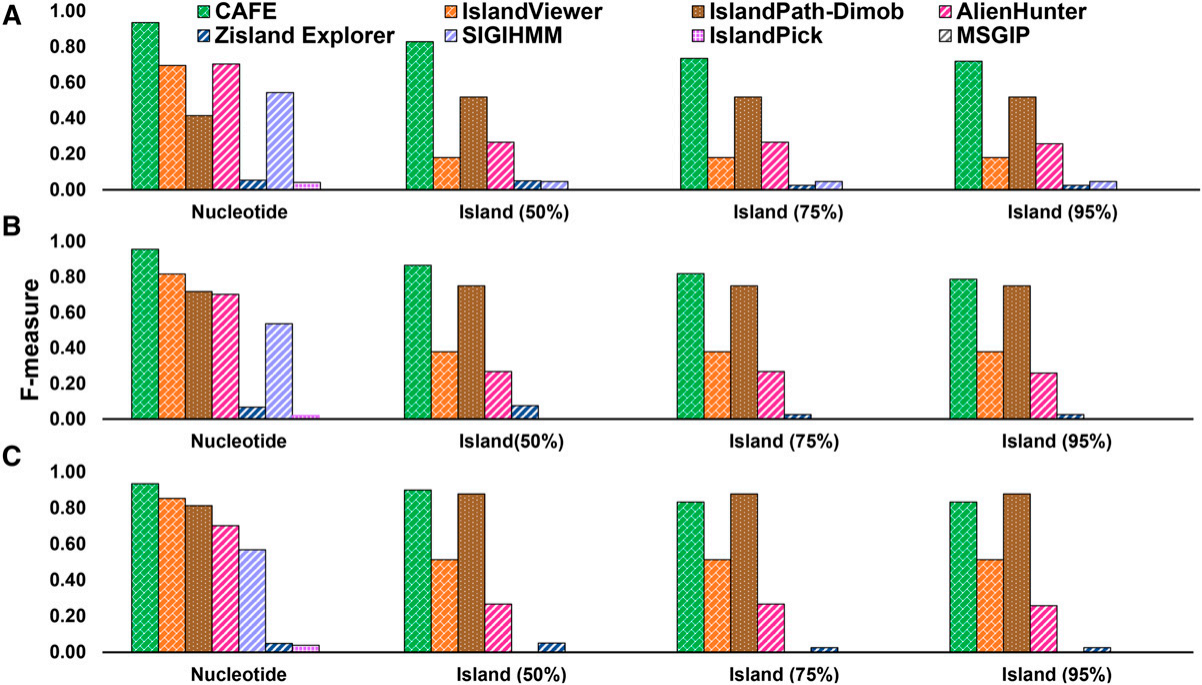
Assessment of genomic island prediction tools on synthetic *Burkholderia cenocepia* genomes. F- measure values (Y-axis; averaged over 5 synthetic genome replicates) are shown for different GI prediction methods and datasets when markers are artificially introduced into A) 25%, B) 50%, and C) 75% of all GIs in the genomes, for nucleotide level, and for island level at different overlap cutoffs (numbers in parenthesis on the X-axis; Z% cutoff means that Z% of a known GI should be identified with the predicted segment (or chain of contiguous segments) in order for the prediction to be deemed a success; see text for further details). Segmentation, contiguous clustering and non-contiguous clustering were performed at thresholds 10^−10^, 10^−13^, and 10^−13^ respectively. Methods based on composition, phylogenetics, and marker gene detection are shown with diagonal brick fill in bars. Bars with a dotted fill show methods using both composition and presence of marker genes for identifying GIs. Methods relying only on sequence composition are shown with upward diagonal lines, and method based only on sequence comparison (IslandPick) is shown with grid.

IslandCafe outperformed other methods by 23–93% in F-measure, 17–94% in Average Performance, and 34–88% in Performance Coefficient on the dataset with 25% marker gene GIs (Table 2, Figure 2A and Table S6), and similarly on the dataset with 50% marker gene GIs (by 14–96% in F-measure, Figure 2B, Table S7) and on the dataset with 75% marker gene GIs (by 9–94% in F-measure, Figure 2C, Table S8). IslandCafe’s MCC was also significantly higher, *e.g.*, 0.93 *vs.* 0.70 of the next best performing method AlienHunter on the dataset with 25% marker gene GIs (Table 2). In general, the performance of marker based methods improved with increasing number of GIs with markers; IslandPath-Dimob’s and IslandViewer’s F-measure for identifying GI nucleotides increased from 0.41 and 0.69 (25% GIs with marker genes) to 0.81 and 0.85 (75% GIs with marker genes) respectively (Figure 2, Table S6-S8). As expected, the performance of the methods based solely on composition did not vary significantly with changes in marker abundance; among these methods, AlienHunter attained the highest nucleotide level F-measure of 0.70 (Figure 2, Tables S6-S8). IslandCafe could attain high sensitivity and specificity, thus outperforming other methods in identifying GI nucleotides in all synthetic datasets with nucleotide-level F-measure values of 0.93, 0.96 and 0.94 for 25%, 50%, and 75% of GIs with marker genes respectively (Figure 2, Tables S6-S8).

**Table 2:** Comparative assessment of genomic island prediction methods in classifying GI and non-GI nucleotides in a synthetic *Burkholderia cenocepia* genome having 25% GIs with marker genes. The values of the performance metrics were obtained by averaging over 5 synthetic genome replicates. Highest value for each performance metric is shown shaded

GI Prediction tool	Recall	Precision	Average Performance	Performance Coefficient	F-measure	MCC
IslandCafe	0.88	0.99	0.94	0.88	0.93	0.92
AlienHunter	0.99	0.55	0.77	0.54	0.70	0.67
IslandViewer	0.59	0.84	0.72	0.53	0.69	0.66
SIGIHMM	0.37	0.99	0.68	0.37	0.54	0.57
DIMOB	0.27	0.95	0.61	0.26	0.41	0.46
Zisland Explorer	0.03	0.12	0.08	0.03	0.05	−0.03
IslandPick	0.02	0.18	0.10	0.02	0.04	0.01
MSGIP	0.00	0.00	0.00	0.00	0.00	0.00

#### Identification of GI in synthetic genomes:

At island level, IslandCafe outperformed other methods at all cutoffs on the dataset with 25% marker gene GIs, bettering IslandViewer and IslandPath-Dimob by 54–65% and 20–31% respectively in F-measure (Figure 2A, Table S6). IslandCafe displayed better performance on the dataset with 50% marker gene GIs as well, outperforming IslandViewer and IslandPath-Dimob by 41–49% and 4–12% respectively in F-measure (Figure 2B, Table S7). On the dataset with 75% marker gene GIs, while IslandCafe outperformed all methods at the 50% cutoff, it was outperformed by IslandPath-Dimob by 5% in F-measure at the 75% and 95% cutoffs (Figure 2C, Table S8). Notably, in all cases, whereas IslandCafe balanced the Recall and Precision very well, other methods produced large differences between Recall and Precision (Table S6-S8).

#### Using real genomes for assessment:

To assess the performance of IslandCafe and other GI prediction tools on genuine genomes, we used our compiled dataset of known GIs; these GIs have previously been used for assessment of GI prediction tools (Jani *et al.* 2016; Soares *et al.* 2016; Wei *et al.* 2017). Although Zisland Explorer (Wei *et al.* 2017) was assessed on *Cronobacter sakazakii* ATCC BAA-894 as well, we did not include it due to the lack of supporting evidence for the reported GIs. This test dataset has a total of 138 GIs, 109 of which harbor markers. As with synthetic genomes, each method was evaluated for its ability to identify GI nucleotides and the GIs themselves as single segments or chains of contiguous segments.

#### Classification of GI and non-GI nucleotides in real genomes:

**Figure 3:**
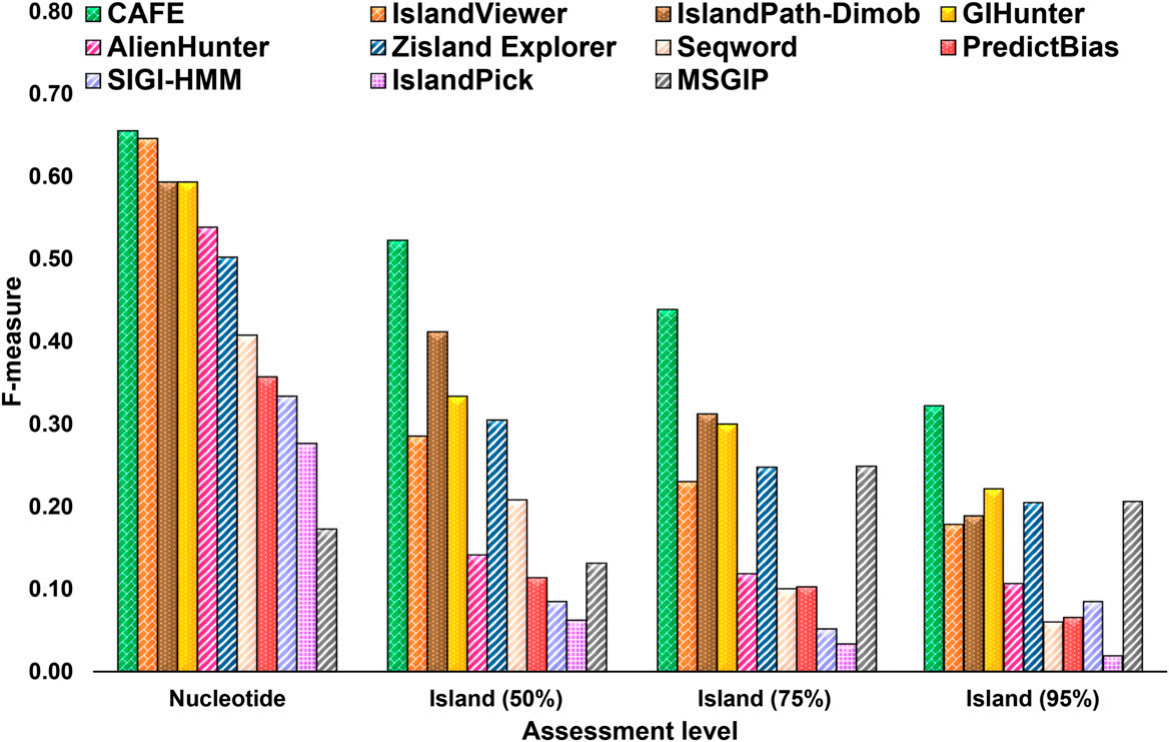
Assessment of genomic island prediction tools in identifying GI nucleotides and GIs in 15 representative bacterial genomes. F-measure values for nucleotide level and island level performance assessment are shown on the Y-axis for different GI prediction methods. Island level F-measure values are shown for different overlap cutoffs (numbers in parenthesis on the X-axis; Z% cutoff means that Z% of a known GI should be identified with the predicted segment (or chain of contiguous segments) in order for the prediction to be deemed a success; see text for further details). Segmentation, contiguous clustering, and non-contiguous clustering were performed at thresholds 2·10^−1^, 10^−5^, and 10^−3^ respectively. Methods based on composition, phylogenetics, and marker gene detection are shown with diagonal brick fill in bars. Bars with a dotted fill show methods using both composition and presence of marker genes for identifying GIs. Methods relying only on sequence composition are shown with upward diagonal lines, and method based only on sequence comparison (IslandPick) is shown with grid.

IslandCafe balanced sensitivity and specificity well (Recall and Precision respectively in Table 3) in identifying the GI borne nucleotides, thus outperforming other methods by up to 49% in F-measure, up to 48% in Average performance, and up to 40% in Performance Coefficient (Table 3, Figure 3). Both IslandCafe and IslandViewer performed comparably, with the former slightly outperforming the latter (by ∼1–3% in overall accuracy assessment) (Table 3). IslandCafe, however, outperformed IslandPath-Dimob by larger margins (by ∼2–7% in overall accuracy assessment, Table 3). GIHunter was found to be most sensitive, identifying ∼87% of GI nucleotides, however it had a precision of only 45%. On the other hand, IslandPath-Dimob had the highest precision of 67%, however, it could identify only 53% of the GI nucleotides. Zisland Explorer and IslandViewer balanced sensitivity and precision (as indicated by small difference between Recall and Precision values), however, both Recall and Precision values were relatively lower for ZislandExplorer (45% and 56% respectively, compared to 71% and 61% by IslandCafe, Table 3).

**Table 3:** Comparative assessment of genomic island prediction methods in classifying GI and non-GI nucleotides in 15 representative bacterial genomes. Highest value for each performance metric is shown shaded

GI Prediction tool	Recall	Precision	Average Performance	Performance Coefficient	F-measure	MCC
IslandCafe	0.71	0.61	0.66	0.49	0.66	0.62
IslandViewer	0.72	0.59	0.65	0.48	0.65	0.59
IslandPath-Dimob	0.53	0.67	0.60	0.42	0.59	0.55
GIHunter	0.87	0.45	0.66	0.42	0.59	0.57
AlienHunter	0.74	0.42	0.58	0.37	0.54	0.50
Zisland Explorer	0.45	0.56	0.51	0.34	0.50	0.46
Seqword	0.35	0.50	0.42	0.26	0.41	0.36
PredictBias	0.81	0.23	0.52	0.22	0.36	0.33
SIGI-HMM	0.24	0.57	0.40	0.20	0.33	0.32
IslandPick	0.19	0.50	0.35	0.16	0.28	0.25
MSGIP	0.23	0.14	0.18	0.09	0.17	0.07

The comparative genomics approach, as in IslandPick, did not perform as well as the composition based approaches, including IslandCafe (Table 3). The performance of this class of methods is contingent upon the genomes selected for comparison and the availability of closely related genomes. Though IslandPath-Dimob that relies on the presence of mobility genes was most precise, it is, however, prone to miss true GIs (*e.g.*, those that may have lost the mobility genes), and therefore suffers from relatively low sensitivity. High sensitivity of GIHunter can be attributed to the large size of its predicted GIs (average size of GIs predicted by GIHunter is 58.2 Kbp compared to 44.9 Kbp by IslandCafe). PredictBias predicted 1268 GIs, highest among all methods (compare with IslandCafe’s 172) and therefore, as expected, has relatively high recall but low precision (Table 3).

Although IslandCafe and IslandViewer performed well in balancing the sensitivity and precision on an aggregated dataset comprised of genomes from 15 species, yielding higher overall accuracy, the performance of GI prediction tools may however vary on individual genomes or chromosomes. The performance of a method may depend on a number of factors, such as, compositional variability including variations in GC-content, availability of closely related genomes, size and amount of GIs in a genome, and perhaps how well a genome is annotated. IslandCafe performed well in identifying nucleotides belonging to GIs in *Vibrio cholerae*, *Streptococcus equi* and *Bordetella petrii* (F-measure and MCC > 0.7) (Table S9). It also performed better than other methods on *Mesorhizhobium loti* (highest values for all accuracy metrics). All GI prediction methods had difficulty finding GI nucleotides in *Salmonella enterica* (highest F-measure value was 0.56 by ZislandExplorer) and *Corynebacterium diptheriae* (highest F-measure value was 0.6 by IslandPath-Dimob; Table S9). These results highlight the complementary strengths of GI prediction methods. Overall, IslandCafe outperformed other GI prediction methods in identifying nucleotides of the known GIs. Furthermore, IslandCafe displayed highest, performance coefficient, F-measure, and MCC for three genomes, more than any other methods.

#### Identification of GIs in real genomes:

At island level, highest accuracy was attained by IslandCafe for all cutoffs. At the 50% cutoff, IslandCafe outperformed other methods by up to 46% in F-measure (Figure 3 and Table 4). IslandCafe was also most precise, outperforming other methods by up to 42% in Precision. IslandCafe outperformed the next best performing method IslandPath-Dimob by 11% in F-measure, bettering both Recall and Precision by 10% and 11% respectively. At 60% Recall, IslandViewer was most sensitive, outperforming IslandCafe by ∼1%, however, its Precision was only 19% compared to 47% of IslandCafe. Of the 442 predictions by IslandViewer, 83 were known GIs at the 50% cutoff. In contrast, of the 172 predictions by IslandCafe, 81 were known GIs. Zisland Explorer predicted fewer GIs than IslandCafe, however, it could identify only 43 knowns GIs (Table 4). IslandCafe outperformed other methods at the 75% and 95% cutoffs as well, with the highest F-measure (Figure 3, Tables S10 and S11), outperforming the next best methods IslandPath-Dimob by 13% and GIHunter by 10% for 75% and 95% cutoffs respectively. At both 75% and 95% cutoffs, IslandCafe was again the most precise method, while PredictBias and IslandViewer were most sensitive (Figure 3, Table S10 and S11).

**Table 4:** Performance assessment of genomic island prediction methods in identifying GIs in 15 bacterial species at the 50% overlap cutoff. At this cutoff, 50% or more of a known GI should be identified with the predicted segment (or chain of contiguous segments) in order for the prediction to be deemed a success (see Tables S9 and S10 for 75% and 95% overlap cutoffs respectively, and the text for further details). Highest value for each performance metric is shown shaded

GI Prediction tool	Recall	Precision	F-measure	Number of GIs Identified	Number of GIs Predicted
IslandCafe	0.59	0.47	0.52	81	172
IslandPath-Dimob	0.49	0.36	0.41	67	187
GIHunter	0.43	0.27	0.34	60	220
Zisland Explorer	0.31	0.30	0.31	43	143
IslandViewer	0.60	0.19	0.29	83	442
Seqword	0.22	0.19	0.21	31	159
AlienHunter	0.51	0.08	0.14	70	847
MSGIP	0.18	0.10	0.13	25	242
PredictBias	0.57	0.06	0.11	78	1268
SIGI-HMM	0.13	0.06	0.09	18	285
IslandPick	0.09	0.05	0.06	13	276

We also assessed the performance of GI prediction tools in localizing GIs in each genome. IslandCafe displayed highest F-measure for six genomes at 50% overlap cutoff as well as at 75% cutoff (Tables S12 and 13), more than any other methods. IslandCafe also had the highest average F-measure (per genome) at 50% and 75% cutoffs (Tables S12 and S13). At 95% cutoff, IslandCafe had the highest F-measure for three genomes and the highest F-measure per genome (Table S14). At this cutoff, IslandViewer had the highest F-measure for six genomes (Table S14). At the island level, IslandCafe thus had highest values of F-measure for up to six genomes out of fifteen, and the highest overall accuracy. Comparative assessment of IslandCafe’s performance at both nucleotide and island levels highlights its strength in identifying GIs as contiguous elements.

Results from the nucleotide and island level assessment demonstrate the overall superior performance of IslandCafe over other methods. Although IslandCafe outperformed IslandViewer slightly in identifying nucleotides of known GIs in real genomes, its performance in identifying known GIs as single segments was substantially better than that of IslandViewer. IslandCafe compared favorably with IslandPath-Dimob as well at both nucleotide and island levels (note that both these programs require the presence of markers in their predictions). Considering that some programs such as IslandViewer take a rather holistic approach by combining several GI prediction methods to raise the accuracy bar, we show here there is still scope for substantial improvement in standalone statistical methods by utilizing biological information to augment their power of discrimination. Notably, our analysis revealed the complementary strengths of different methods, which is further elaborated in the Discussion section below.

### Analysis of novel genomic islands

We examined GIs predicted by IslandCafe that do not overlap with known GIs. IslandCafe identified 72 novel GIs in 15 bacteria we studied (Table S15). These islands display atypical composition and were identified within clusters that were either marker-enriched or displayed unusual phyletic pattern, providing multiple lines of evidence in support of their possible horizontal acquisition. Of the 72 novel GIs identified by IslandCafe, 20 have marker genes such as transposase, integrase or phage related genes (Table S15). The compositional, phylogenetic and marker analyses performed by IslandCafe suggest that these “false positives” are likely true positives, and accuracy rendered by IslandCafe is likely much higher. This may apply to other methods as well, particularly those that rely on multiple lines of evidence. However, as revealed in this study, almost all existing methods have difficulty recovering the GI structure (see island level accuracies in Figures 2 and 3, Table 4 and, Tables S10 and S11). With a relatively much better performance in island recovery, IslandCafe offers new opportunities for researchers to investigate the novel GIs, perhaps alongside novel predictions by other promising methods such as IslandPath-Dimob.

## Discussion

Although top-down, recursive segmentation is a powerful tool for genome analysis, adapting this approach to delineate GIs necessitates parameter optimization, robust cluster merger, and elimination of spurious predictions. In this study, we show that the combining diverse evidence helps in minimizing the sensitivity of parametric methods on parameter setting. Significance thresholds that result in optimal performance of the segmentation and clustering often vary between bacterial taxa, however, by incorporating GI specific feature enrichment and phyletic distribution analyses within this framework, we identified parameters that were globally applicable on a diverse set of bacterial species in delineating GIs with high accuracy (segmentation threshold: 2∙10^−1^, contiguous clustering threshold: 10^−5^, and non-contiguous clustering threshold:10^−3^). Thus suggests that once clusters are reliably populated within a broad range of stringent thresholds, feature enrichment and phyletic distribution analyses suffice to group native clusters of apparently variable composition and thus enable robust detection of GIs. This could reflect on overall performance as is apparent in IslandCafe’s higher single accuracy metric values in identifying known GIs from 15 representative genomes in comparison to segmentation-clustering; the values for nucleotide-level Average Performance, Performance Coefficient, F-measure, and MCC were 0.66, 0.49, 0.66, and 0.62 respectively for the former, while 0.55, 0.34, 0.50, and 0.47 respectively for the latter (Tables 1 and 3). In genome-wise comparison as well, segmentation-clustering at genome specific optimal parameter setting could not perform better than IslandCafe at its single, universal parametric setting on a majority of genomes (Table 1 and Table S8). Although genome specific optimal parameter setting is hard to realize for yet uncharacterized genomes and the quest will always be for a universal parameter setting (the “default setting”) that yields the best overall performance, future efforts could focus on organism or species specific parameter setting where feasible to further enhance the performance and take the GI prediction to even greater heights.

Since compositional disparity is a strong indicator of recent HGT (Vernikos & Parkhill 2006), a host of methods exploiting the compositional or codon usage biases have been developed to localize alien genes or GIs (de Brito *et al.* 2016; Jani *et al.* 2016; Waack *et al.* 2006; Wei *et al.* 2017). Methods or visualizations tools that search for signals, such as, the remnants of GI integration in the recipient genome and markers or features associated with GIs have also been developed (Hsiao *et al.* 2003; Pundhir *et al.* 2008). Attempts have also been made to combine the sensitive compositional approach with the conservative signal sensor approach (Langille & Brinkman 2009). Our approach to detect GIs via clustering and enrichment stands out among the current methods in its ability to perform unbiased detection of GIs through grouping of genomic segments of similar origin based on composition and enrichment. GIs that are weakly atypical or those that lack identifying markers are difficult to identify; clustering allows their identification via association with GIs from the same source. Marker-deficient GIs are prone to be missed by the conventional approaches. These GIs could group with marker-enriched GIs originating from a donor source within our proposed framework, and therefore, could be identified based on association. Marker-deficient GIs from a source, if allowed to be grouped and analyzed together, as in IslandCafe, may even display significantly greater enrichment than the native clusters. The predicted segments with only one or two markers are likely to be missed by conventional methods that base their predictions on abundance, unlike IslandCafe that bases its inference on grouping and enrichment. An example is shown in Table S16, where 20 segments (annotated GI 1 – GI 20) from the backbone genomes of three donors, namely, *D. thermolithotrophum*, *F. nucleatum*, and *M. agalactiae* were inserted into the backbone genome of *A. baumannii*. *D. thermolithotrophum* and *F. nucleatum* contributed 7 GIs each, while *M. agalactiae* contributed 6 GIs; half of these GIs lacked any markers. Application of optimized IslandCafe to this synthetic *A. baumannii* genome with 20 GIs revealed a robust grouping of GIs from a donor source, both with and without markers. All seven GIs from *D. thermolithotrophum* were assigned to a single cluster (Cluster ID 2, Table S16). The two *F. nucleatum* clusters (Cluster IDs 6 and 7) were also not contaminated with GIs from other sources. All 6 GIs from *M. agalactiae* were assigned to a single cluster (Cluster ID 8). This demonstrates the ability of IslandCafe to identify marker devoid GIs via their association with GIs with markers through a pipeline of segmentation, clustering, and enrichment.

We further emphasize the augmentation of generic attributes of this pipeline through incorporation of a phylogenetic module in posteriority to enable identification of marker deficient GI clusters. By incorporating both enrichment and phylogenetic modules within an integrated framework of segmentation and clustering, we have strived to develop an approach that can work well on just sequenced, yet uncharacterized genomes. Though segmentation and clustering identify the native genome well, yet in several clusters, with some very small. These smaller native clusters show atypical composition for reasons other than HGT, however, these clusters are likely not enriched in GI specific features and/or lack unusual phyletic pattern. Therefore, IslandCafe takes a two-pronged approach (enrichment and phyletic distribution) to address this. First, it attempts to minimize false positives by allowing merger of non-enriched clusters into the largest (native) cluster and in parallel, it attempts to minimize false negatives by ensuring that those non-enriched clusters that display unusual phyletic pattern are not merged. This augments the precision substantially, without a significant decrease in recall, resulting in significant improvement in overall accuracy (*e.g.*, F-measure). The power of IslandCafe thus lies in its ability to precisely delineate GIs due to the segmentation approach, identify GIs devoid of markers by their association with marker-enriched GIs (in clusters), and identify GIs displaying unusual phyletic pattern by association again (in clusters). Whereas many predicted GIs are expected to display atypical composition *and* marker enrichment or unusual phyletic pattern, some with only atypical composition could also be identified because of association, deciphered via clustering. IslandCafe, in principle, is thus more precise than segmentation and clustering approach, while retaining the generic attributes of this approach. Our results indeed support this (compare IslandCafe’s Recall and Precision of 0.71 and 0.61 with the respective 0.72 and 0.39 by Segmentation and Clustering; Tables 1 and 3).

As different methods often test different hypotheses, this may lead to non-converging predictions from these programs. Reconciliation of divergent sets of predictions is a challenge, however, this also indicates that no single method alone can address this problem and a holistic approach that combines the complementary strengths of different methods must be explored. We suggest to the readers, based on our comparative analysis, to collate the predictions from IslandCafe and IslandViewer into a single set for their genome of interest. Both programs base their predictions on multiple lines of evidence thereby reducing the likelihood of generating false positives. As they clearly showed complementarity by outperforming each other substantially on different sets of genomes (Tables S9, S12-S14), union of their predictions could substantially augment the sensitivity with only marginal or negligible additions of false positives. We further recommend, based on IslandCafe’s demonstrated ability to more efficiently recover the GI structure (Figures 2 and 3), that IslandCafe’s predictions be relied upon where there are disagreements between the two programs on the structures of the predicted GIs. In summary, we demonstrated the robustness and universality of the simple approach implemented in IslandCafe by assessing on synthetic test datasets and on a set of well-understood genomes sampled from different bacterial lineages. Overall, IslandCafe was found to be most accurate among the GI prediction methods, however, none of the methods could outperform all others on all genomes considered in this study. Our results reveal the complementarity of different approaches and suggest usage of compositional, phylogenetic, and functional or structural features in concert to comprehensively catalog GIs. We show here that enriching a statistical framework with biological information is a step forward in more robust delineation of GIs. Such frameworks will become even more relevant as more information encoded within genomes is deciphered and utilized within these frameworks. Future efforts could also focus on developing integrative approaches by exploiting the complementarity of different methods, which enables boosting the sensitivity without compromising the specificity (see, for example, Azad & Lawrence 2005).
